# New Developments in Prokinetic Therapy for Gastric Motility Disorders

**DOI:** 10.3389/fphar.2021.711500

**Published:** 2021-08-24

**Authors:** Michael Camilleri, Jessica Atieh

**Affiliations:** Clinical Enteric Neuroscience Translational and Epidemiological Research (CENTER), Division of Gastroenterology and Hepatology, Mayo Clinic, Rochester, MN, United States

**Keywords:** aprepitant, domperidone, erythromycin, functional dyspepsia, gastroparesis, ghrelin, prucalopride, relamorelin

## Abstract

Prokinetic agents amplify and coordinate the gastrointestinal muscular contractions to facilitate the transit of intra-luminal content. Following the institution of dietary recommendations, prokinetics are the first medications whose goal is to improve gastric emptying and relieve symptoms of gastroparesis. The recommended use of metoclopramide, the only currently approved medication for gastroparesis in the United States, is for a duration of less than 3 months, due to the risk of reversible or irreversible extrapyramidal tremors. Domperidone, a dopamine D2 receptor antagonist, is available for prescription through the FDA’s program for Expanded Access to Investigational Drugs. Macrolides are used off label and are associated with tachyphylaxis and variable duration of efficacy. Aprepitant relieves some symptoms of gastroparesis. There are newer agents in the pipeline targeting diverse gastric (fundic, antral and pyloric) motor functions, including novel serotonergic 5-HT_4_ agonists, dopaminergic D_2/3_ antagonists, neurokinin NK_1_ antagonists, and ghrelin agonist. Novel targets with potential to improve gastric motor functions include the pylorus, macrophage/inflammatory function, oxidative stress, and neurogenesis. In the current review, we discuss the use of pharmacological approaches with potential to enhance motor functions in the management of gastroparesis.

## Introduction: Definitions and Currently Available Prokinetic Treatments

Gastroparesis is characterized by upper gastrointestinal symptoms including nausea, vomiting, early satiety, postprandial fullness, bloating, and upper abdominal pain, as well as slow gastric emptying of solids in the absence of gastric outlet or intestinal obstruction. In clinical practice, the most common etiologies of gastroparesis are diabetes mellitus, idiopathic, iatrogenic (post-surgical or medication), diseases affecting the neural control arising in the brain and spinal cord (such as Parkinson disease, which may also be associated with effects of dopaminergic agents), and diseases that damage intrinsic nerves or smooth muscle, often as a result of tissue infiltration (such as scleroderma) or muscle degeneration (as in amyloidosis).

Prokinetic agents are medications that amplify and coordinate gastrointestinal muscular contractions (Acosta and Camilleri, 2015), including coordination between different segments of the gut, thereby enhancing propulsion of intra-luminal contents. Some prokinetics are active in selective areas of the gastrointestinal tract, whereas the activity of other prokinetics is more generalized and reflects the location of receptor targets of the pharmacological agents. In the current review, we discuss the use of prokinetics in the management of gastric motility disorders.

In most countries, only two medications are approved or available for the treatment of gastroparesis: metoclopramide and domperidone. Both agents are antagonists at dopamine-2 (D_2_) receptors. The effect of the endogenous transmitter, dopamine, is to inhibit the release of acetylcholine (ACh), and this results in decrease in motility of the stomach and proximal small bowel (Tonini et al., 2004). These inhibitory effects of endogenous dopamine are reversed by D2 receptor antagonists. In general, metoclopramide and domperidone were equally effective for relief of symptoms, although central nervous system adverse effects were more common with metoclopramide (Patterson et al., 1999; Camilleri et al., 2013). Current guidelines recommend liquid formulation metoclopramide, 5 to 10 mg orally, 30 min before meals and at bedtime in patients with gastroparesis. Higher doses are not recommended in order to avoid side effects. A new intra-nasal formulation of metoclopramide has also been approved by the Food and Drug Administration (FDA) of the United States.

Domperidone is available for physician prescription through FDA’s program for Expanded Access to Investigational Drugs (https://www.fda.gov/drugs/investigational-new-drug-ind-application/how-request-domperidone-expanded-access-use). The recommended starting dose of domperidone is 10 mg, t.i.d., which could be increased (if necessary) to 20 mg, t.i.d., and at bedtime. Domperidone has been associated with cardiac dysrhythmias which led to its removal in Europe from the over-the-counter space to availability only by prescription. It is generally recommended that domperidone be avoided if the corrected QTc interval on the patient’s electrocardiogram is > 470 ms in males and >450 ms in females.

An off-label prokinetic approach for gastroparesis involves the use of inhibitors of acetyl cholinesterase. Neostigmine is a short-acting agent that was shown to accelerate gastric emptying of liquids in patients who are critically ill and have delayed gastric emptying; this was associated with induction of an irregular increase in gastric and duodenal contractility (Bortolotti et al., 1995; Lucey et al., 2003). Neostigmine has a short duration of action, is administered parenterally by slow intravenous or intramuscular injection. Therefore, its use is limited to the hospital setting with electrocardiogram monitoring because of the potential to induce vagotonia and bradycardia.

Pyridostigmine has a longer duration of action; it is not approved for treatment of gastroparesis; it is available in liquid or tablet formulation and, based on clinical experience, it is used at doses up to 60 mg, t.i.d. There are, as yet, no clinical trials documenting its clinical efficacy in gastroparesis. In an open-label case series, pyridostigmine was beneficial in relief of symptoms in children with such upper gastrointestinal motility problems as chronic intestinal pseudo-obstruction, delayed small bowel transit accompanying gastroparesis, and chronic constipation associated with failure to thrive. Effective dosing ranged between 0.25 and 2.0 mg/kg/day (Manini et al., 2017).

The serotonergic 5-HT_4_ receptor agonist, cisapride, was associated with symptomatic benefit in patients with gastroparesis based on short-or medium-term placebo-controlled trials (Corinaldesi et al., 1987; Camilleri et al., 1989; Richards et al., 1993a), and on long-term, open-label studies (Abell et al., 1991). Although cisapride accelerated gastric emptying, it did not necessarily enhance glycemic control, over the long-term (Braden et al., 2002). Cisapride is no longer available in most countries due to withdrawal as a result of cardiovascular concerns (cardiac arrhythmias due to inhibition of the human ether-à-go-go-related gene [hERG] potassium channel).

Other medications available in a few countries, clebopride, cinitapride, and mosapride, are not reviewed in detail here in view of the relatively weak evidence of efficacy in gastroparesis. There are no controlled trials of clebopride (D2 antagonist) in gastroparesis other than in dyspepsia with radiologically delayed gastric emptying, a criterion not currently accepted for gastroparesis (Bavestrello et al., 1985). Cinitapride (a 5-HT_1_ and 5-HT_4_ agonist and 5-HT_2_ antagonist) was superior to placebo in a parallel design study of 19 patients with dyspepsia associated with postprandial distress and mildly to moderately delayed gastric emptying (Portincasa et al., 2009). Mosapride (a 5-HT_4_ agonist) enhanced gastric emptying in gastroparesis associated with treatment with interferon, but had no significant effects on symptoms (Kawamura et al., 2012).

There is, therefore, significant and unmet clinical need to develop new prokinetic agents for gastric motility disorders. Over the past few years, it has been appreciated that the upper gastrointestinal symptoms that are consistent with gastroparesis may arise from diverse gastric motor dysfunctions that constitute potential targets for pharmacological agents, thus, expanding the spectrum of therapeutic approaches.

### Gastric Motor Dysfunctions

There are three dominant motor dysfunctions that can result in diverse manifestations or symptoms: gastric emptying, gastric accommodation, and pyloric dysfunction. It is relevant to note that, in patients with upper gastrointestinal symptoms, there are about 25% of patients with delayed gastric emptying, about 25% with impaired gastric accommodation, and about 25% with the combination of both gastric motor dysfunctions (Park et al., 2017; Chedid et al., 2019a). In addition, among patients with gastroparesis, a subset of those with antral hypomotility also has evidence of pylorospasm (Mearin et al., 1986).

In this article, new developments in prokinetic therapy for these motility disorders are reviewed. Prior to exploring the pharmacological approaches using prokinetics, it is useful to review the overall principles regarding the methods used to measure those gastric motor functions as they are used in pharmacodynamic assessment of the therapeutic approaches.

Identification of disorders of gastric emptying requires an accurate gastric emptying test. The optimal gastric emptying diagnostic tests typically involve measurements at standard times (e.g., 0, 0.5, 1, 2, 3, and 4 h) over 4-h by scintigraphy or by stable isotope breath tests. The symptoms associated with retardation of gastric emptying are nausea, vomiting, and upper abdominal bloating, but pain is not a quintessential symptom of delayed gastric emptying. A significant relationship has been demonstrated between the acceleration of gastric emptying and the improvement of symptoms. Thus, using a meta-regression analysis, it was demonstrated that acceleration in gastric emptying T_1/2_ of 20.4 min was associated with a1-unit reduction in the severity of symptoms. This analysis was based on standardized mean difference in order to account for differences in the measurements of symptoms between studies (Vijayvargiya et al., 2019). Normal values and performance characteristics of the scintigraphic test have been published (Szarka et al., 2008; Camilleri et al., 2012). Disorders of gastric emptying can be ameliorated by targeting specific receptors, including serotonergic 5-HT_4_, dopamine D_2/3_, and neurokinin NK_1_ receptors.

Disorders of gastric accommodation are typically associated with postprandial distress syndrome, a component of functional dyspepsia. In fact, approaches to enhance postprandial accommodation have been associated with reduced symptoms of functional dyspepsia, for example, by using the serotonergic 5-HT_1A_ agonist, buspirone, or by using acotiamide, an antagonist of acetylcholinesterase and antagonist of presynaptic M_1_ and M_2_ muscarinic receptors. These muscarinic receptors are involved in inhibition of acetylcholine release. Therefore, by antagonizing those receptors and by inhibiting acetylcholinesterase, acotiamide leads to an increased local level of acetylcholine, which is an excitatory transmitter in the enteric nervous system and parasympathetic nerve pathways.

There are currently three direct and one indirect measurements of gastric accommodation. The three direct methods are: first, single-photon emission computerized tomography (SPECT) imaging (Bouras et al., 2002), second, measurement of proximal gastric volume by barostat, whereby the pressure of air within an infinitely compliant polyethylene balloon is clamped (maintained constant by an electronic pump aspirating or infusing air), and the continuous monitoring of the intra-balloon volume provides a measurement of gastric tone. A third method is intraluminal high resolution manometry in the proximal stomach (Tack et al., 1998; Carbone et al., 2017). In addition, an indirect measurement of gastric accommodation can be obtained through ingestion of a nutrient drink at constant rate until the maximum tolerated volume (MTV) is reached]; this measurement assesses gastric sensation (Tack et al., 2003). However, in addition, it provides an indirect measure of accommodation if the MTV is less than ∼750 kilocalories, since there is a linear correlation between the MTV and gastric accommodation volume measured by a barostat when the MTV is below 750 kcal (Tack et al., 2003). There have been attempts to use two-dimensional imaging of the area of the proximal stomach immediately after food ingestion to estimate gastric accommodation; however, these measurements were subsequently found to be inaccurate relative to three-dimensional imaging and the 2-D imaging method therefore requires further validation (Orthey et al., 2018a; Orthey et al., 2018b; Chedid et al., 2019b).

Disorders of pyloric function are difficult to assess noninvasively and two approaches are available, requiring intraluminal measurements. These are antropyloroduodenal manometry and the Endoflip (endoscopic functional lumen imaging probe) device. The former uses closely-spaced manometric sensors to measure the pressure profile and identifies pyloric activity by the combination of phasic and tonic contractions, as well as the combination of antral and duodenal phasic pressure activity in the manometric tracing (Nelson et al., 2016). The Endoflip device is a longer (8 or 16 cm) probe consisting of 16 paired impedance planimetry electrodes mounted on a catheter and located within a balloon that is filled with a conductive fluid (typically distended with 40–50 ml fluid); excitation electrodes at either end of the balloon generate a low electric current. The impedance electrodes measure voltage and, using the voltages, the device calculates the cross-sectional areas (CSA) using Ohm’s Law (resistance = voltage/current) at each electrode interval. A solid-state pressure transducer is located at the distal end of the balloon. Thus, by measuring the pressure simultaneously with the CSA, it is possible to calculate a distensibility index (Vosoughi et al., 2020).

### Novel Pharmacotherapies: Current State of Evidence

Symptoms may result from diverse pathophysiological disorders including accelerated or slow emptying, reduced gastric accommodation, gastric dysrhythmias, or duodenal mechanisms. It has therefore been proposed that future treatment of gastric motor dysfunctions and related symptoms should be based on identified pathophysiology or “actionable biomarkers” that is putative mechanisms that are associated with induction of the symptoms suggesting gastroparesis and that can be normalized with specific treatments, such as treatments targeting5-HT_4_, dopamine D_2_ and D_3_, and NK_1_ receptors. The available and investigational prokinetic agents discussed below for gastric motility disorders are summarized in Table 1.

**TABLE 1:** Current and investigational prokinetic drugs for gastric motility disorders.

Drug name	Disease	Effect on gastric motor function	GP symptoms	Ref. #
**5-HT4 receptor agonist**				
Prucalopride	IG and DG	↑ GE	Improved	Carbone et al. (2019)
Velusetrag	IG and DG	↑ GE	Improved	Kuo et al. (2021a)
Felcisetrag	IG and DG	↑ GE	Not studied	(Chapman et al., 2021; Chedid et al., 2021)
Tegaserod	FD	↑ GA	Mixed effects	(Vakil et al., 2008; Tack et al., 2010)
**D2/3 receptor antagonist**				
Trazpiroben	IG and DG	↑ volume to fullness, No Δ in GE	Improved	Kuo et al. (2021b)
**Ghrelin receptor agonist**				
Relamorelin	DG	↑ GE, ↑ antral contractions	Improved	(Shin et al., 2013a; Shin et al., 2013b; Lembo et al., 2016; Nelson et al., 2016; Camilleri et al., 2017; Camilleri et al., 2020)
**Muscarinic M1/2 receptor antagonist**				
Acotiamide	FD	↑ GE and GA	Improved	Kusunoki et al. (2012)
**Motilin receptor agonist**				
Erythromycin	IG and DG	↑ GE, ↑ fundic and antral contractions, ↓ pyloric contractions	Improved	(Janssens et al., 1990; Catnach and Fairclough, 1992; Richards et al., 1993b; Parkman et al., 1995)
Azithromycin	Gastroparesis	↑ GE	Not studied	Larson et al. (2010)
Clarithromycin	FD	↑ GE	Not studied	Bortolotti et al. (1999)
**NK1 receptor agonist**				
Aprepitant	IG and DG	↑ GA, No Δ in GE	Improved	(Jacob et al., 2017; Pasricha et al., 2018)
Tradipitant	IG and DG	Not studied	Improved	Carlin et al. (2021)
**Opioid antagonists [non-selective (NS) or peripherally active (PAMORA)]**				
Naloxone [NS]	FD and IG	No Δ in GE	Not studied	Narducci et al. (1986)
MNTX [PAMORA]	opioid-induced gastric delay	No Δ in GE	Not studied	Wong et al. (2010)
Naloxegol [PAMORA]	opioid-induced gastric delay	No Δ in GE	Not studied	Halawi et al. (2018)
**Phosphodiesterase-5 Inhibitor**				
Sildenafil	Gastroparesis with uremia	No Δ in GE	Not studied	Dishy et al. (2004)

Abbreviations: DG, diabetic gastroparesis; FD, functional dyspepsia; GA, gastric accommodation; GE, gastric emptying; GP, gastroparesis; IG, idiopathic gastroparesis; MNTX, methylnaltrexone; PAMORA, peripherally active μ‐opioid receptor antagonist.

### Novel 5-HT_4_ Receptor Agonists Targeting Gastric Emptying

Several “new generation” 5-HT_4_ receptor agonists are selective for 5-HT_4_ receptors withouthERG effects (De Maeyer et al., 2006; Tack et al., 2012); these include prucalopride, velusetrag, naronapride, and felcisetrag.

Prucalopride is approved by the European Medicines Agency (EMA) and the FDA for the treatment of chronic constipation. In a randomized, placebo-controlled, cross-over study involving 34 patients (28 idiopathic, six diabetic) with gastroparesis, patients received prucalopride, 2 mg once daily, or placebo for 4 weeks, with a 2-weeks washout between treatments. Prucalopride was efficacious in relieving symptoms based on total Gastroparesis Cardinal Symptom Index, subscales of nausea/vomiting, fullness/satiety, and bloating/distention, as well as improvement in the overall Patient Assessment of Upper Gastrointestinal Disorders-Quality of Life score (Carbone et al., 2019). Similarly, velusetrag, was reported to be efficacious in the treatment of diabetic and idiopathic gastroparesis (Kuo et al., 2021a).

Intravenously administered felcisetrag significantly accelerated gastric emptying, small bowel transit and colonic transit compared to placebo in patients with gastroparesis with previously confirmed delayed gastric emptying. Felcisetrag was well tolerated (Chedid et al., 2021). In a previous double-dummy, parallel-group, randomized trial, felcisetrag (TAK-954), administered to mechanically ventilated patients with enteral feeding intolerance defined as gastric residual volume ≥200 ml, led to a greater proportion of patients with normal gastric retention compared to four doses of 10 mg metoclopramide (Chapman et al., 2021).

Velusetrag and felcisetrag (TAK-954) had no significant effects on coronary tone (demonstrated in dog, pig or human coronary arteries) or cardiac rhythm (hERG channel potassium conductance) or platelet function), or other off-target actions (Beattie et al., 2013). Felcisetrag has high affinity (pK(i) = 9.4) for human recombinant 5-HT_4c_ receptors and >2,000-fold selectivity for those receptors compared to 78 other receptors (including all other 5-HT receptors, several non-5HT receptors), transporters or ion channels tested (Beattie et al., 2011).

Another potential mechanism to enhance neuromuscular function in the stomach is an anti-inflammatory effect that may facilitate vagal stimulation. This has been demonstrated with the 5-HT_4_ agonist, prucalopride, which modified responses of T2helper cells and shortened post-operative ileus (Matteoli et al., 2013; Bosmans et al., 2017; Stakenborg et al., 2019). There is evidence that macrophages that are not derived from circulating monocytes, are resident in the gut, are distinct from CD206 (also called M2) macrophages, and can impact enteric nervous system function (De Schepper et al., 2018). This anti-inflammatory mechanism may be relevant since some animal models have inflammation and oxidative stress-induced damage to the enteric nervous system and pacemaker cells (discussed below).

### Targeting Sensations With D_2_/D_3_ and NK_1_ Antagonists

For upper gastrointestinal disorders associated with increased gastric sensation, such as functional dyspepsia, the dopaminergic D_2_/_3_ antagonist, trazpiroben (TAK-506) administered for 1 week significantly increased the volume to fullness during a nutrient drink test, compared to baseline (Kuo et al., 2021b). Moreover, in a placebo-controlled trial, the NK_1_ receptor antagonist, aprepitant, improved multiple symptoms of gastroparesis including nausea (Pasricha et al., 2018). These beneficial effects may reflect the known effects of NK1 receptor antagonists on the vomiting center in the brainstem, akin to the action associated with reduced chemotherapy-induced emesis. Another potential mechanism for the symptomatic benefit may be related to increased fasting and accommodation volumes of the stomach without deleterious effect on gastric emptying, which has been demonstrated in healthy controls (Jacob et al., 2017). The novel NK_1_ receptor antagonist, tradipitant, improved several symptoms of gastroparesis in a 4-weeks, randomized, controlled trial (Carlin et al., 2021). The symptomatic benefit was most marked in patients with vomiting among the baseline symptoms; it was interesting to note that improvement of nausea was associated with improvement of all the other symptoms evaluated.

### Ghrelin Receptor Agonist

Ghrelin is predominantly located in the stomach. It is an appetite-stimulating 28 amino acid hormone. Administration of a pharmacological dose of recombinant human ghrelin increased proximal gastric tone through central and peripheral effects (Peeters, 2003; Tack et al., 2006), and in some studies, it also accelerated stomach emptying in patients with gastroparesis [reviewed in ref. (Camilleri et al., 2009).].

**FIGURE 1:**
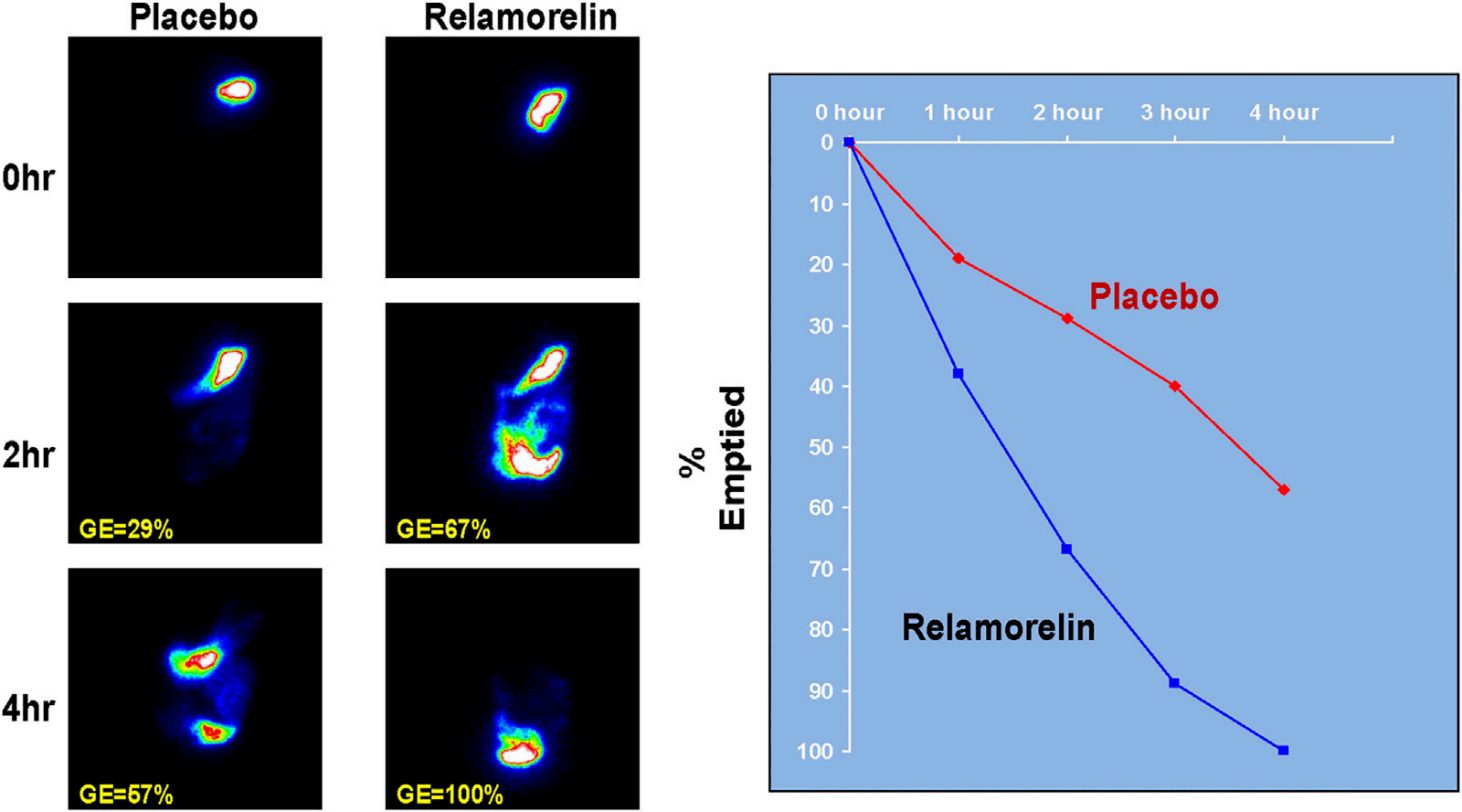
Effect of relamorelin on gastric emptying in a patient with type 1 diabetes with gastroparesis. Aadapted from ref. 51, Shin A, et al. Clin Gastroenterol Hepatol 2013; 11:1,453–1,459.

**FIGURE 2:**
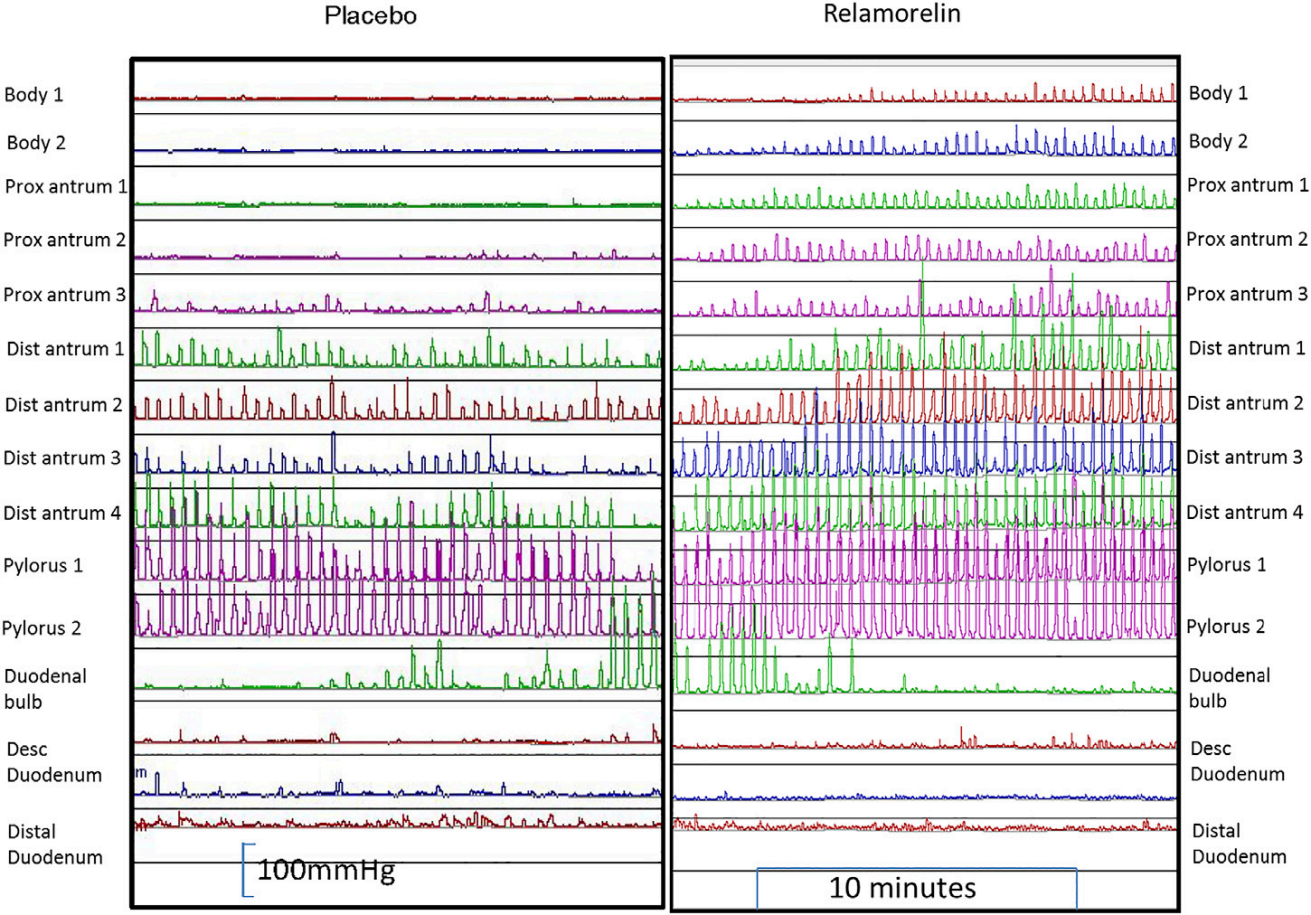
Effect of relamorelin on antral motility in a healthy subject. Reproduced with permission from ref. 29, Nelson A, Camilleri M, et al. Neurogastroenterol Motil 2016; 28:1,705–1,713.

A synthetic pentapeptide ghrelin receptor agonist, relamorelin, is 15–130 times more potent than natural ghrelin (Van der Ploeg et al., 2014). At a dose of 100 mg subcutaneously (s.c.), relamorelin accelerated gastric emptying of solids Figure 1 in patients with either type 1 or type 2 diabetes mellitus with prior documentation of delayed gastric emptying (Shin et al., 2013a; Shin et al., 2013b). Relamorelin also increased distal antral contraction frequency without impeding gastric accommodation or altering postprandial satiation in healthy volunteers Figure 2 (Nelson et al., 2016), which differentiates its effects from those of the macrolide antibiotic, erythromycin. In phase 2A and 2B, randomized, controlled trials in patients with diabetic gastroparesis, relamorelin improved clinical symptoms and appears to be safe, other than the induction of (typically postprandial) hyperglycemia which is attributable to the acceleration of gastric emptying (Lembo et al., 2016; Camilleri et al., 2017; Camilleri et al., 2020). It has therefore been recommended that proactive steps should be taken to control postprandial glycemia in diabetics receiving relamorelin (Camilleri et al., 2020).

### Motilin Receptor Agonists

The most common motilin receptor agonists are the macrolide antibiotics that stimulate gastrointestinal motilin receptors, especially gastric receptors. Erythromycin improves gastric emptying and transiently improves symptoms before there is downregulation of the motilin receptor, typically in about 4 weeks after onset of therapy (Richards et al., 1993b; Thielemans et al., 2005), manifesting as tachyphylaxis or reduced treatment efficacy. One of the attractions of erythromycin is that it stimulates fundic and antral contractions, while inhibiting pyloric contractility (Janssens et al., 1990; Catnach and Fairclough, 1992; Parkman et al., 1995). The current recommended dose for hospitalized patients with gastroparesis is 1.5–3 mg/kg (by i.v. infusion over 45 min) every 6–8 h, and 125 mg b.i.d., orally for outpatient gastroparesis management for a few weeks. The liquid formulation is often preferred in order to enhance pharmacokinetics in patients with marked delay in gastric emptying. Side effects experienced with erythromycin treatment include abdominal pain, nausea, and diarrhea. Caution should be taken when erythromycin is co-administered with agents that alter or are metabolized by cytochrome P450 (CYP) 3A4 (e.g., diltiazem or verapamil or domperidone) because the drug interactions may be associated with risk for sudden cardiac death (Ray et al., 2004).

Azithromycin and clarithromycin are other macrolides that accelerate gastric emptying (Bortolotti et al., 1999; Larson et al., 2010). There are no randomized, placebo-controlled trials to assess efficacy. Use of these agents should be balanced with the potential of tachyphylaxis due to downregulation of the target receptors, cardiac risk as mentioned above, or development of bacterial resistance to these useful antimicrobial agents.

### Targeting the Gastric Fundus

Mechanistic studies showed that acotiamide enhanced gastric accommodation and gastric emptying of a liquid meal (Kusunoki et al., 2012) and improved symptoms in patients with functional dyspepsia (Matsueda et al., 2012). Some 5-HT_4_ receptor agonists also enhance gastric accommodation, such as tegaserod in dyspeptics with normal gastric emptying (Tack et al., 2010). This provides a rationale for their use in functional dyspepsia. However, two randomized controlled trials that studied tegaserod in patients with functional dyspepsia demonstrated mixed effects on dyspepsia symptoms (Vakil et al., 2008).

Recent data using simultaneous measurement of gastric accommodation and emptying of a solid egg meal suggests that there is a direct relationship between the degree of gastric accommodation and the gastric emptying lag duration and T_1/2_, suggesting that, in some patients, impaired emptying may result from excessive gastric accommodation with delayed movement of solid food from the fundus to the antrum of the stomach (Wang et al., 2021). This observation suggests that stimulation of the proximal stomach with reduced gastric accommodation may actually enhance gastric emptying in patients with gastroparesis.

It is not surprising, therefore, that erythromycin is associated with marked acceleration or dumping of food from the stomach since, as a motilin receptor agonist and stimulant of cholinergic mechanisms, it enhances both fundic contraction as well as antral motor function, thereby having a dual effect on mechanisms associated with increased longitudinal axial forces in the antrum in healthy participants and patients with dysmotilities and acceleration of gastric emptying (Surrenti et al., 1996; Coulie et al., 1998; Liau et al., 2001). Further studies on the potential of fundic and antral stimulants to improve gastric symptoms would be of considerable interest.

### Targeting the Pylorus

It is increasingly recognized that patients on long-term opioid medications may present with gastroparesis (Hasler et al., 2019). The mechanisms associated with the effects of opiates or opioid medications are reviewed elsewhere (Camilleri and Sanders, 2020). Opioids can induce pyloric dysfunction in addition to inhibition of antral motor function, both of which contribute to delayed gastric emptying. It is, therefore, relevant to assess whether targeting the pylorus or inhibiting the effects of opioids might be a therapeutic approach for gastric emptying delay attributed in part to pyloric dysfunction.

Although the classical pharmacological approach to treating the pylorus in gastroparesis involves botulinum toxin injection and there is open-label experience to suggest efficacy especially with higher dose injections (Coleski et al., 2009), two placebo-controlled trials did not demonstrate efficacy (Arts et al., 2007; Friedenberg et al., 2008).

Two pharmacological approaches have been pursued to reverse pyloric dysfunction in gastroparesis. One approach is the use of sildenafil (Watkins et al., 2000), a phosphodiesterase-5 inhibitor which mimics the effect of nitric oxide by increasing intracellular cGMP. A reduced expression of neuronal nitric oxide synthase in the pylorus of diabetic mice was reversed by treatment with insulin and by sildenafil. However, in gastroparesis associated with uremia, there was no significant effect of sildenafil on gastric emptying (Dishy et al., 2004).

A second approach to reverse pyloric contractility involves use of opioid antagonists. In an older study, naloxone did not stimulate gastric emptying in healthy subjects or in patients with gastric hypomotility associated with functional dyspepsia or idiopathic gastroparesis (Narducci et al., 1986). Two studies have tested the potential of peripherally active μ‐opioid receptor antagonists (PAMORA) in the setting of codeine-induced delay in gastric emptying in opioid-naïve healthy participants. At the doses approved for treatment of chronic opioid‐induced constipation (OIC), short-term administration of methylnaltrexone (s.c. 0.30 mg/kg) or naloxegol (25 mg) daily did not inhibit the retardation of gastric emptying induced by codeine in healthy, opioid-naïve volunteers (Wong et al., 2010; Halawi et al., 2018).

As stated above, there is evidence of pyloric relaxation by erythromycin by stimulating the inhibitory nerves of the pylorus; however, the long-term effects of erythromycin on the pylorus are unclear and they have not been studied extensively.

### Targeting M_2_ Macrophages and Oxidative Stress

Gastroparesis may also result from abnormal function of enteric mechanisms which may be targeted by macrophage-based immune dysregulation, as demonstrated in diabetic mice with delayed gastric emptying (Cipriani et al., 2018). Reduced pacemaker cells [interstitial cells of Cajal (ICCs), and platelet-derived growth factor receptor alpha (PDGFRα) fibroblast-like cells] and numbers of nitrergic neurons and CD206 positive macrophages have been reported in some studies in patients with idiopathic or diabetic gastroparesis (Grover et al., 2011; Grover et al., 2012; Grover et al., 2017), though the ICC results were not confirmed in patients with idiopathic gastroparesis (Bernard et al., 2014; Herring et al., 2018). Damage to the pacemaker cells may occur because of depletion of anti-inflammatory resident M_2_ macrophages expressing heme oxygenase-1 (HO_1_), allowing oxidative stress to damage the pacemaker cells or enteric nerves, as evidenced from animal models of gastroparesis (Choi et al., 2008; Cipriani et al., 2018). However, hemin, an heme oxygenase (HO_1_) inhibitor, failed to reverse delayed gastric emptying in a randomized, controlled trial in humans. Unfortunately, the pharmacokinetics of the hemin in that trial were insufficient to conclusively test whether countering oxidative stress can restore normal ENS function and gastric emptying (Bharucha et al., 2016).

### Potential Pharmacological Promotion of Neuronal Cell Differentiation

As in other tissues, there is a dynamic balance in the enteric nervous system between cell loss by apoptosis, phagocytosis of dead cells by resident macrophages, and restoration of new cells from stem cells. Similarly, in the enteric nervous system, the loss of neurons is replenished by neurogenesis precursor cells that behave like stem cells and are prominent in the submucosal zone and in the muscular layers (Kulkarni et al., 2017). It has been shown, *in vitro,* that a selective estrogen receptor β (ERβ) agonist, LY3201, stimulated glial-to-neuron cell differentiation. It also increased recovery of neurons in the damaged myenteric plexus in two *in vivo* models of enteric neuronal damage in mice, specifically the damage resulting from administration of a high-fat diet, or the serosal application of the cationic detergent, benzalkonium chloride (D'Errico et al., 2018).

The potential for neurogenesis and nerve growth factors to restore normal propulsion has been demonstrated in both animal and human studies. In rats, exogenous brain-derived neurotrophic factor (BDNF) increased myoelectric activity and peristalsis in the gastrointestinal tract and colon (Chai et al., 2003; Grider et al., 2006). In addition, exogenous recombinant human BDNF and neurotrophin-3 were shown to accelerate gastrointestinal and colonic transit in healthy human volunteers and in patients with constipation respectively (Coulie et al., 2000). Thus, neurogenesis has the potential to improve enteric nervous system function in patients with gastroparesis.

## Conclusion

There has been extensive research into the effects of diverse classes of medications targeting different pathophysiological mechanisms including defective contraction or coordination of the stomach manifesting with symptoms and objective retardation of gastric emptying. The findings reported augur well for the development of effective treatments for gastroparesis. These include immune (such as macrophage) modulation, reversal of oxidative stress, direct pharmacological therapies targeting pivotal receptors without inducing adverse effects, and endoscopic pyloromyotomy. Further validation studies and approval by regulatory agencies should lead to opportunities to resolve the significant unmet clinical need in patients with gastroparesis.
